# P-176. Detecting Evidence for Bystander Selection by Comparing Trends in Antibiotic Resistance among Infection Causing Bacteria in a Large Global AMR Database

**DOI:** 10.1093/ofid/ofae631.381

**Published:** 2025-01-29

**Authors:** Rashmi Madhukar, Quentin Leclerc, Gwen Knight

**Affiliations:** University of Michigan, Ann Arbor, Michigan; Pasteur Institute, Paris, Ile-de-France, France; London School of Hygiene and Tropical Medicine, London, England, United Kingdom

## Abstract

**Background:**

Unintentional exposure to antibiotics meant for the treatment of infections caused by other bacteria promote the selection of antibiotic resistant strains in off-target organisms in a phenomenon called bystander selection (BS). Some of these organisms could subsequently cause drug resistant infections or spread to others, promoting antibiotic resistant bacteria in the population. The importance of BS as a driver for antimicrobial resistance (AMR) at the global level is not well understood. We conducted an ecological time trends study to identify and compare trends over time of different clinically relevant bacteria-antibiotic pairs to detect evidence for BS globally and in the United States.

Schematic Representation of the Development of Bystander Selection

**Figure:**
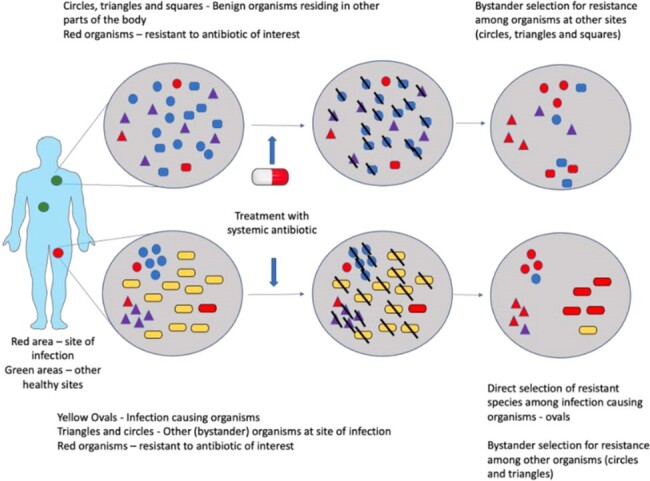

**Methods:**

We identified global and US trends in resistance using the Cochran-Armitage and Mann-Kendall tests. We compared trends in resistance using Pearson’s correlation coefficient. Among bacteria pairs with highly correlated trends in resistance, we eliminated pairs that belong to the same genus or commonly colonize the same niche, where horizontal genome transfer may explain the high degree of correlation, to arrive at bacteria pairs that were most likely experiencing BS.

**Figure:**
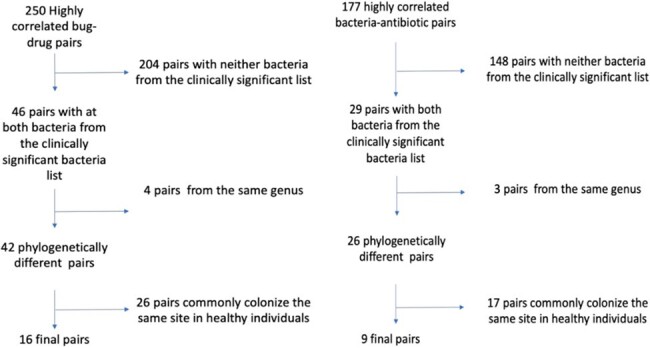

Figure showing how we arrived at the 16 and 9 highly correlated antibiotic-bacteria pairs that have the most likely experienced bystander selection globally and in the United States.

**Results:**

We found 16 and 9 bacteria pairs respectively from our global and United States analysis that most likely experienced BS. Trends in resistance of *Acinetobacter baumannii* to carbapenems and cefepime were highly correlated with members of the family *Enterobacteriace* and *Psuedomonas*. In the United States, rates of levofloxacin resistance among *Staphylococcus aureus* were significantly correlated with *Strepococcus pneumoniae*.

Figure showing the final 16 bacterial pairs from the global analysis in whom bystander selection was the most likely cause for significantly correlated trends in antimicrobial resistance.

**Figure:**
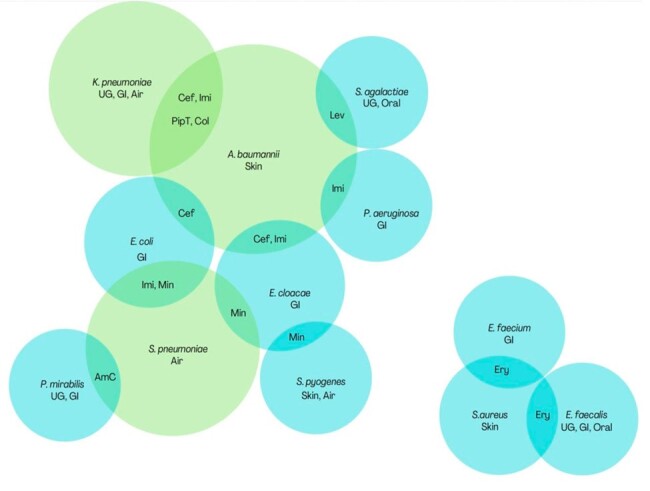

Each bubble represents one bacterial species. Intersection between 2 bubbles represents highly correlated trends in resistance between those 2 bacterial species to the antibiotics mentioned in the overlapping area. The size of the bubble represents the total number of correlations for that bacterium. Bubbles of bacterial species with 4 or more correlations are depicted in green.

**Conclusion:**

To the best of our knowledge this is the first study to find evidence for BS driving the development of AMR in a global AMR surveillance database. Our findings suggest that judicious use of carbapenems for the treatment of *Pseudomonas, E*. *coli,* K. *pneumoniae* and *Enterobacter* infections could help address carbapenem resistance in *A. baumannii*. This work highlights the need to study antimicrobial resistance using a systems approach as opposed to studying the development of resistance in single bacteria-antibiotic pairs.

Figure showing the final 9 bacterial pairs from the US specific analysis in whom bystander selection was the most likely cause for significantly correlated trends in antimicrobial resistance.

**Figure:**
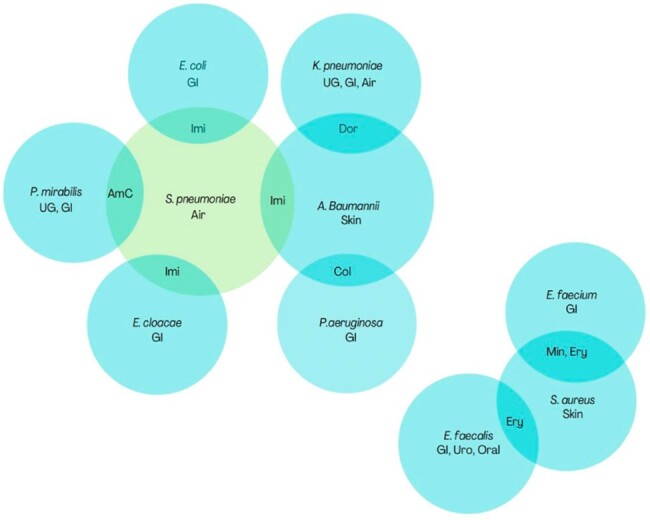

Each bubble represents one bacterial species. Intersection between 2 bubbles represents highly correlated trends in resistance between those 2 bacterial species to the antibiotics mentioned in the overlapping area. The size of the bubble represents the total number of correlations for that bacterium. Bubbles of bacterial species with 4 or more correlations are depicted in green.

**Disclosures:**

**All Authors**: No reported disclosures

